# Supplementary material for: Bounding cross-shelf transport time and degradation in Siberian-Arctic land-ocean carbon transfer
Source: Nat Commun. 2018 Feb 23;9:806. doi: 10.1038/s41467-018-03192-1 (PMC5824890; doi:10.1038/s41467-018-03192-1)
Supplement: Supplementary file 1 — Supplementary Information [file 41467_2018_3192_MOESM1_ESM.pdf]

Supplementary Information

Bounding cross-shelf transport time and degradation in Siberian-Arctic land-ocean carbon transfer

Bröder et al.

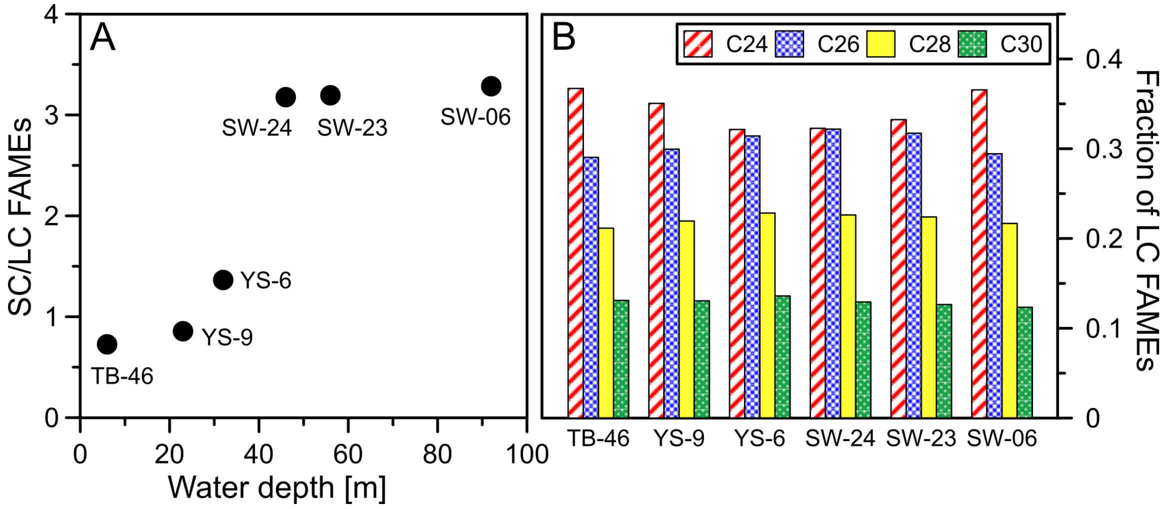

Supplementary Figure 1. Changes in FAME chain lengths along the transect. (A) The ratio of short- to long-chain (SC/LC) FAMEs increases with water depth along the Laptev Sea transect due to a growing contribution of marine organic matter to the bulk OC. SC: C14-C23, LC: C24-C30. (B) Relative proportions of the homologues with different carbon-chain lengths (C24, C26, C28, C30) are relatively constant for the same sediment samples (increasing water depth from left to right).

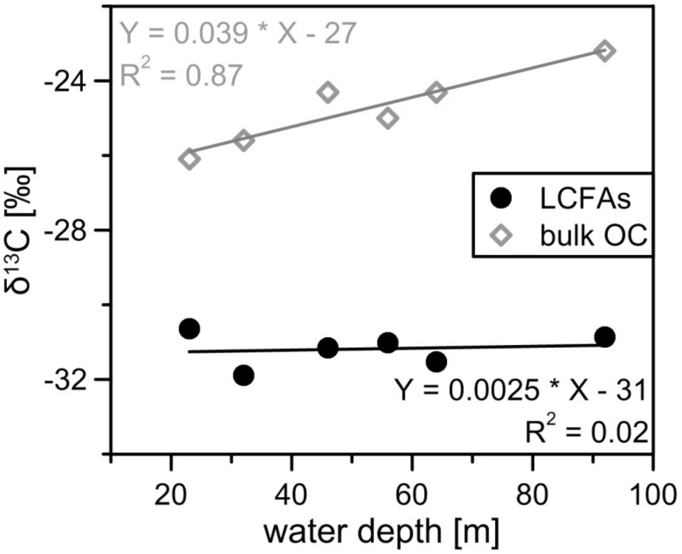

Supplementary Figure 2. Stable carbon isotope values for bulk OC (open grey diamonds) and long-chain *n*-fatty acids (LCFAs, closed black circles) display different behaviors with increasing water depth. Increasing $\delta^{13}C$ values for bulk OC can be explained by an increasing proportion of marine OC, whereas the constantly low values for the LCFAs confirm their terrestrial provenance.

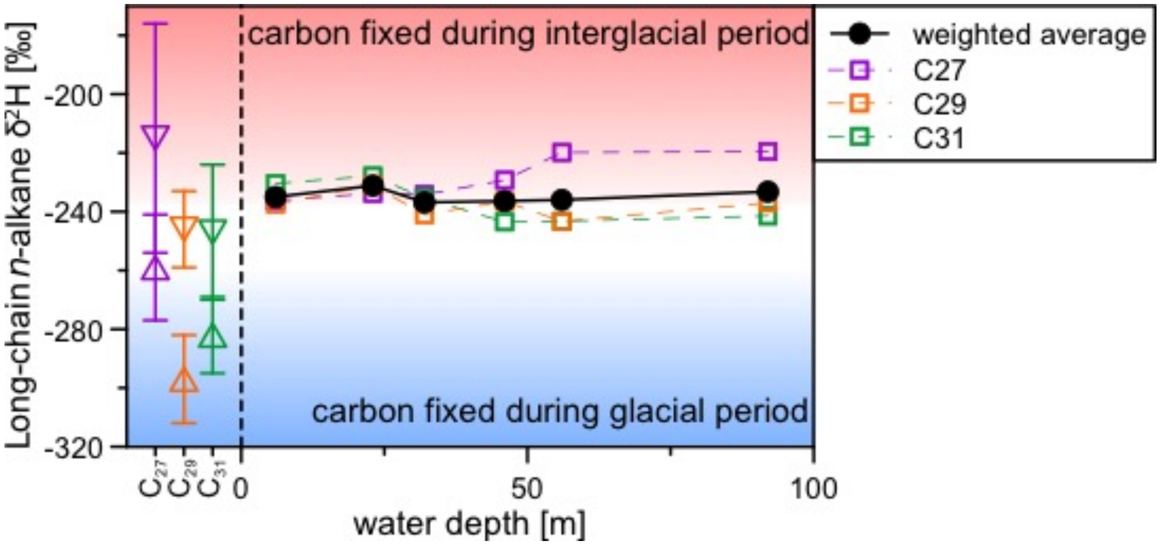

Supplementary Figure 3. Isotopic ratios of stable hydrogen isotopes δ^2H of long-chain *n*-alkanes for selected stations across the Laptev Sea shelf (part of a larger dataset[1]) and for Ice Complex Deposit and active layer endmember values (up- and downward pointing triangles, respectively) as in Vonk et al.[1]. Shaded also the approximate ranges for carbon fixation during glacial (cold, blue) and interglacial (warm, red) periods as in Zech et al.[2] (Tumara Paleosol Sequence in Northeast Siberia, 63°36′ N 129°58′ E). The concentration-weighted average of carbon-chain lengths 27, 29 and 31 does not display a significant trend with water depth and thereby supports the hypothesis of similar terrOC sources along the transect.

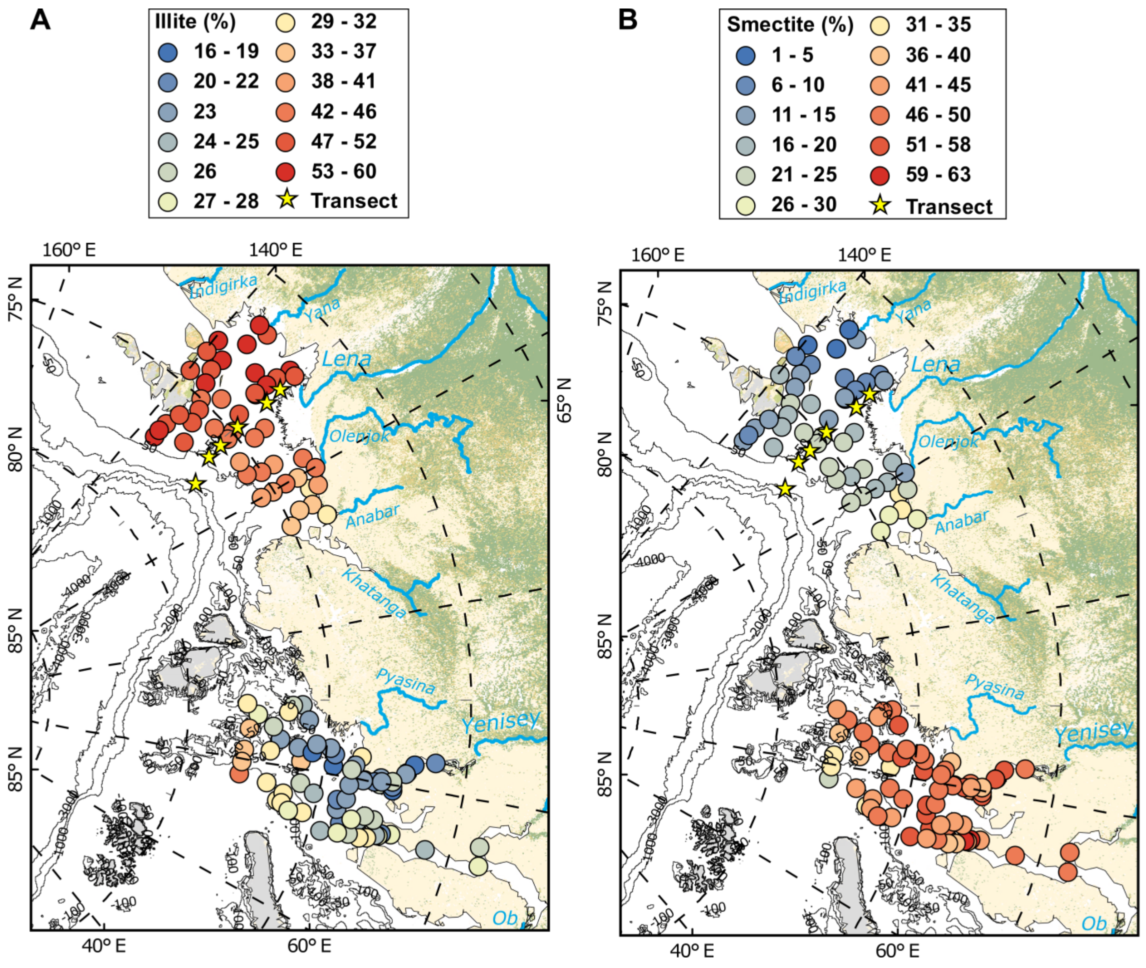

Supplementary Figure 4. Data compilation on the relative contributions of clay minerals Illite (A) and Smectite (B) in surface sediments from the Laptev and Kara seas[3–5]. Sampling stations for this study are marked with yellow stars. Clay minerals supplied by Khatanga, Ob and Yenisey rivers are enriched in smectite (average smectite content in the Khatanga River of up to 84 %[5] due to weathering of flood basalts in the Poutorian Mountains[4]), whereas the Lena River suspended sediments are composed mainly of illite (sedimentary Mesozoic and Paleozoic rocks) with an average of ca. 54 %[5].The surface mineral clay distribution thus confirms that the Lena River is the dominant sediment source along the shelf transect. The underlying map was made with ArcGIS 10 using the latest version of the bathymetric grid IBCAO 3.0[6].

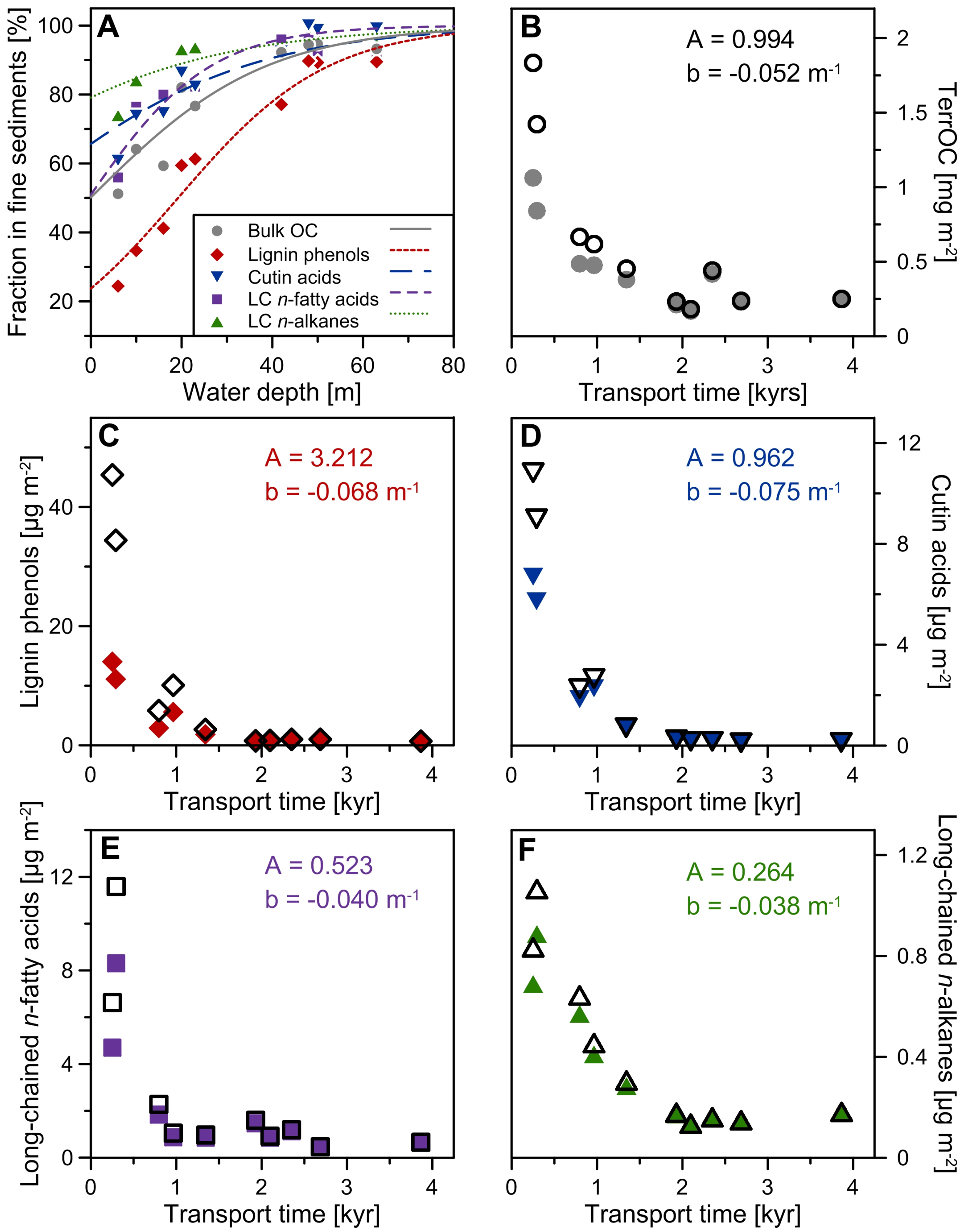

Supplementary Figure 5. Correction for hydrodynamic sorting during transport.

Data in panel (A) are from Tesi et al.[7], where the organic carbon and terrestrial biomarker distributions were measured in different sediment density, size and settling velocity

fractions for surface sediments from the East Siberian Arctic Shelf. Here we used the obtained relationship between proportion OC/biomarker in the fine fraction and water depth (fitted logistic functions: $\boldsymbol{y} = \mathbf{1}\left(+\ \boldsymbol{A} \times \boldsymbol{e}^{-\boldsymbol{bx}}\ ^{-\mathbf{1}}\right)$, see parameters A and b for each dataset in Panels B-F) to correct bulk values for terrOC/biomarker loadings. For each sample, the fraction in fine sediments (<63 μm) is calculated according to the water depth at the sampling location. Loadings before (black open symbols) and after (colored symbols) correcting for hydrodynamic sorting during transport: (B) TerrOC, (C) lignin phenols, (D) cutin acids, (E) long-chain (LC) *n*-fatty acids, (F) LC *n*-alkanes. Values for bulk sediments are from Bröder et al.[8]. The correction was performed using the relationship between water depth and fraction biomarker in the fine sediments from Panel A.

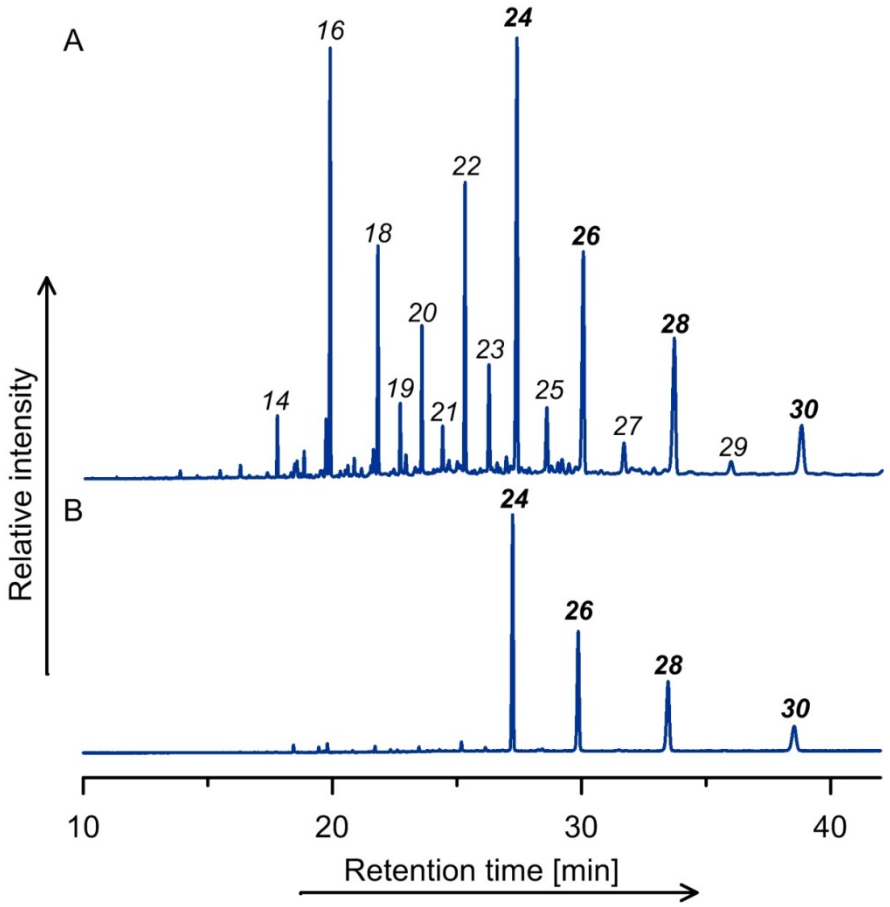

Supplementary Figure 6. Total ion current gas chromatogram of the FAME fraction of surface sediment sample TB-46 close to the Lena River delta (A) before and (B) after preparative capillary gas chromatography. The FAMEs are marked with numbers indicating the carbon chain lengths.

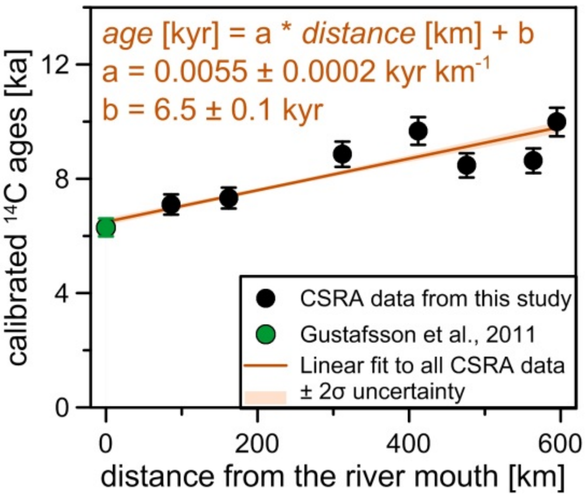

Supplementary Figure 7. Calibrated radiocarbon ages of long-chain *n*-fatty acids vs distance, using the station closest to the Lena River delta (N-8 from Gustafsson et al.,[9]) as the reference point. A similar good fit as with water depth was obtained (R^2 = 0.78, $p < 0.05$). However, water depth was used instead as it was less biased of choosing a starting point.

Station ID	Latitude	Longitude	Distance from N-8	uncal. CSRA age	±	NOSAMS Receipt #	SiO_2	Al_2O_3	CaO
	° N	° E	km	kyrs BP	kyrs BP		wt %	wt %	wt %
SW-06	77.142	127.378	595	9.16	0.13	135373	72	12.6	1.7
SW-14	76.894	127.798	564	8.03	0.19	135372	71.3	12.5	1.5
SW-23	76.171	129.333	476	7.91	0.09	133037	68.9	13.6	1.4
YS-4	75.987	129.984	450	-	-	-	63.8	15.1	1.3
SW-24	75.599	129.558	412	8.91	0.10	133038	62.5	15.4	1.2
YS-6	74.724	130.016	312	8.22	0.04	133039	62.1	16.1	1.3
YS-9	73.366	129.997	162	6.65	0.03	135374	70.8	14	1.3
YS-13	71.968	131.701	75	-	-	-	61.6	17.4	0.8
YS-14	71.630	130.050	40	-	-	-	69.6	15	1.6
TB-46	72.700	130.180	86	6.46	0.08	133041	67.9	15.2	1.8
N-8	71.955	129.535	0	5.50	0.10	-	-	-	-

Supplementary Table 1. Sampling locations, radiocarbon data and mineral composition for the sediment samples from the Laptev Sea. SiO_2, Al_2O_3 and CaO data were published in Bröder et al.[8]. Data for station N-8 was published in Gustafsson et al.[9].

	**TerrOC** (kyr^{-1})	**Lignin phenols** (kyr^{-1})	**Cutin acids** (kyr^{-1})	**LC *n*-alkanes** (kyr^{-1})	**LC *n*-fatty acids** (kyr^{-1})
Data points <1.5 kyr	8.1±5.0	9.3±4.0	3.7±2.4	8.5±3.7	10±12
All data points	2.4±0.6	2.8±0.2	2.6±0.1	1.9±0.4	4.0±0.9

Supplementary Table 2. A comparison of degradation rate constants computed for a subset of the data points with transport times of less than 1.5 kyr to the degradation rate constants obtained for the entire dataset. The degradation rate constants for the subset with shorter transport times are on average a factor of three higher, but poorly constrained.

Station ID	TOC	SA	$\delta^{13}C$ of TOC	±	TerrOC	±	Lignin phenols	±	Cutin acids	±	LC *n*-fatty acids	±	LC *n*-alkanes	±
	mg g^{-1}	m^2 g^{-1}	‰	‰	mg m^{-2}	mg m^{-2}	ug m^{-2}	ug m^{-2}	ug m^{-2}	ug m^{-2}	ug m^{-2}	ug m^{-2}	ug m^{-2}	ug m^{-2}
SW-06	7.6	14.9	-23.2	0.15	0.25	0.06	0.68	0.10	0.12	0.02	0.67	0.13	0.18	0.04
SW-14	8.9	19.4	-24.3	0.15	0.23	0.06	0.98	0.15	0.12	0.02	0.46	0.09	0.15	0.03
SW-23	15.8	21.7	-25.0	0.15	0.42	0.10	0.93	0.14	0.17	0.02	1.19	0.24	0.16	0.03
YS-4	13.4	31.4	-24.8	0.15	0.22	0.05	0.79	0.12	0.16	0.02	0.92	0.18	0.13	0.03
SW-24	10.7	37.0	-24.3	0.15	0.17	0.04	0.71	0.11	0.21	0.03	1.59	0.32	0.18	0.04
YS-6	18.6	31.6	-25.6	0.15	0.38	0.10	2.33	0.35	0.63	0.09	0.97	0.19	0.31	0.06
YS-9	13.1	16.9	-26.1	0.15	0.48	0.12	8.34	1.25	2.21	0.33	1.05	0.21	0.45	0.09
YS-13	18.9	23.5	-25.9	0.15	0.49	0.12	4.65	0.70	1.81	0.27	2.28	0.46	0.64	0.13
YS-14	19.1	11.4	-26.2	0.15	0.77	0.19	22.51	3.38	5.89	0.88	11.58	2.32	1.07	0.21
TB-46	25.8	12.0	-26.5	0.15	0.94	0.23	28.69	4.30	6.83	1.03	6.62	1.32	0.83	0.17

Supplementary Table 3. Organic carbon composition of sediment samples from the Laptev Sea. Total organic carbon (TOC), surface area (SA) and carbon isotopes as in Bröder et al.[8]. Biomarker loadings from Bröder et al.[8] have been corrected for hydrodynamic sorting during transport according to relationship between water depth and fraction terrOC/biomarker in the fine sediments obtained from data by Tesi et al.[7]. See also Supplementary Figure 6.

Station ID	Purity	Sample type	Depths used for CSIA	Shell found in layer	^{14}C age	NOSAMS Receipt #
SW-06	97.1 %	Core	0-4 cm	3-4 cm	>Modern	129812
SW-14	93.5 %	Core	0-4 cm	3-4 cm	>Modern	135375
SW-23	98.7 %	Core	0-3 cm	2-3 cm	>Modern	129814
SW-24	98.4 %	Core	0-3 cm	2-3 cm	>Modern	129816
YS-6	98.0 %	Core	0-3 cm	-	-	-
YS-9	97.3 %	Grab	surface	-	-	-
TB-46	98.2 %	Grab	surface	-	-	-

Supplementary Table 4. Purity of the isolated C24, C26, C28 and C30 FAMEs (composites), sediment sample type and radiocarbon results for collected shells. For sediment cores the depths from core top are specified, which were used for compound-specific isotope analysis (CSIA) in this study. For all but YS-6 shells from the lower part of the section were dated at NOSAMS. Their modern ages confirmed the assumption that the top 3-4 cm of each core were indeed part of the mixed layer. For YS-6, no shells were found, but unpublished ^{210}Pb data revealed a linear sedimentation rate of ~2 mm yr^{-1}. The top 3 cm then correspond to ~15 yr, which is much smaller than the uncertainties of the compound-specific radiocarbon ages.

Supplementary References

1. Vonk, J. E. *et al.* Distinguishing between old and modern permafrost sources in the northeast Siberian land–shelf system with compound-specific δ2H analysis. *Cryosph.* **11,** 1879–1895 (2017).
2. Zech, R., Huang, Y., Zech, M., Tarozo, R. & Zech, W. High carbon sequestration in Siberian permafrost loess-paleosols during glacials. *Clim. Past* **7,** 501–509 (2011).
3. Rossak, B. T., Kassens, H., Lange, H. & Thiede, J. Clay Mineral Distribution in Surface Sediments of the Laptev Sea : Indicator for Sediment Provinces , Dynamics and Sources. in *Land-Ocean Systems in the Siberian Arctic: Dynamics and History* (eds. Kassens, H. et al.) **2,** 587–599 (Springer-Verlag, 1999).
4. Schoster, F., Behrends, M., Muller, R., Stein, R. & Washner, M. Modern river discharge and pathways of supplied material in the Eurasian Arctic Ocean: Evidence from mineral assemblagesand major and minor element distribution. *Int. J. Earth Sci.* **89,** 486–495 (2000).
5. Dethleff, D., Rachold, V., Tintelnot, M. & Antonow, M. Sea-ice transport of riverine particles from the Laptev Sea to Fram Strait based on clay mineral studies. *Int. J. Earth Sci.* **89,** 496–502 (2000).
6. Jakobsson, M. *et al.* The International Bathymetric Chart of the Arctic Ocean (IBCAO) Version 3.0. *Geophys. Res. Lett.* **39,** 1–6 (2012).
7. Tesi, T., Semiletov, I., Dudarev, O., Andersson, A. & Gustafsson, Ö. Matrix association effects on hydrodynamic sorting and degradation of terrestrial organic matter during cross-shelf transport in the Laptev and East Siberian shelf seas. *J. Geophys. Res. Biogeosciences* **121,** 731–752 (2016).
8. Bröder, L. *et al.* Fate of terrigenous organic matter across the Laptev Sea from the mouth of the Lena River to the deep sea of the Arctic interior. *Biogeosciences* **13,** 5003–5019 (2016).
9. Gustafsson, Ö., Van Dongen, B. E., Vonk, J. E., Dudarev, O. V. & Semiletov, I. P. Widespread release of old carbon across the Siberian Arctic echoed by its large rivers. *Biogeosciences* **8,** 1737–1743 (2011).
